# Blood Pressure Profiles and Cognitive Function from Adulthood to Old Age: Chasing a Golden Middle Way?

**DOI:** 10.3390/jcm10153243

**Published:** 2021-07-23

**Authors:** Rita Del Pinto, Davide Grassi, Raffaella Bocale, Francesco Carubbi, Claudio Ferri, Giovambattista Desideri

**Affiliations:** 1 Department of Life, Health and Environmental Sciences, University of L’Aquila, 67100 L’Aquila, Italy; rita.delpinto@univaq.it (R.D.P.); davide.grassi@univaq.it (D.G.); fcarubbi@asl1abruzzo.it (F.C.); claudio.ferri@univaq.it (C.F.); 2 Division of Endocrine Surgery, “Agostino Gemelli” School of Medicine, University Foundation Polyclinic, Catholic University of the Sacred Heart, 00198 Rome, Italy; raffaella.bocale@policlinicogemelli.it

**Keywords:** hypertension, hypotension, dementia, cognitive impairment, antihypertensive medications

## Abstract

With the demographic shift toward advanced ages, it is imperative to understand the biological mechanisms behind common, disabling age-related diseases such as cognitive impairment in its mild form to overt dementia. Hypertension, a major cardiovascular risk factor, is epidemiologically linked to vascular and Alzheimer-type dementia, with possible mechanisms being atherosclerotic macro- and microvascular damage leading to neuronal cell death, as well as proinflammatory events responsible for neurodegeneration. Nevertheless, there is currently a knowledge gap as to which population to target, what the diagnostics test, and how to manage early pathogenic events in order to prevent such a dramatic and disabling condition. While clinical trials data support the benefit of active BP control with antihypertensive medications on the risk of future cognitive impairment, hypotension appears to be related to accelerated cognitive decline in both the fit and the cognitively frail elderly. Dedicated, technologically advanced studies assessing the relation of BP with dementia are needed to clarify the pathophysiological mechanisms in the association before a tailored preventive, diagnostic, and therapeutic approach to one of the most widespread modern medical challenges becomes a reality.

## 1. Introduction

With the progressive aging of the population and the parallel increase in the burden of age-related diseases, a clear understanding of the factors involved in the maintenance of cognitive competence and self-sufficiency throughout life carries relevant socio-economic implications. A variable degree of cognitive deficit up to full-blown dementia commonly occurs after prolonged exposure to major cardiovascular risk factors, including high blood pressure (BP) [1].

Consistent evidence indicates that hypertension control protects against cognitive deterioration and dementia through BP reduction, with possible additive benefits deriving from pleiotropic effects of certain antihypertensive medications [2]. In parallel, iatrogenic orthostatic hypotension has been related to a more rapid cognitive decline in the elderly [3]. 

With the redefinition of BP targets during antihypertensive treatment toward lower goals and the demographic shift toward advanced ages [4], it is paramount to understand the biological mechanisms underlying the association between BP profile and cognitive performance for a proper definition of effective preventive measures and clinically oriented guidelines. In this narrative review, we summarize lights and shadows in the relation between BP and cognitive performance, with a focus on the elderly.

## 2. Hypertension Control and Neuroprotection

Converging evidence indicates that cardiovascular diseases and related risk factors play an important role in the etiology of both vascular and non-vascular (i.e., Alzheimer-type) dementia [5,6,7,8]. As for hypertension, there is concordant evidence of an association between exposure and subsequent development of cognitive impairment and dementia. Specifically, early adulthood and midlife hypertension were found to be associated with increased risk of later cognitive impairment and dementia [7,9,10,11]. 

In terms of early exposure to high BP, in the Coronary Artery Risk Development in Young Adults (CARDIA) Study, which included 3381 adults aged 18 to 30 years (mean age: 25.1 ± 3.6 years) who were followed-up for 25 years, greater cumulative exposure to elevated systolic and diastolic BP (>120/80 mmHg) was associated with worse cognition in midlife (mean age: 50.2 ± 3.6 years) [7]. Similarly, a retrospective cohort study of 8845 participants of a health maintenance organization who underwent health evaluations between the ages of 40 and 44 and whose 30-year health outcomes were ascertained showed that hypertension was associated with a 24% increase in risk of dementia in late life (adjusted hazard ratio (HR): 1.24, 95% CI 1.04–1.48) [10]. Data from the third generation of the Framingham Heart Study further indicate that asymptomatic vascular brain injury can be detected even in young adults (mean age: 39.2 ± 8.4 years) in linear association with systolic BP [12]. Altogether, these data underscore the need for specifically designed studies assessing the long-term impact of hypertension treatment in young populations.

With reference to exposure to high BP at intermediate ages, a cohort study of 499 individuals of same year of birth who were followed for over 35 years found that high and increasing BP from early adulthood into midlife (43–53 years) was associated with increased white matter hyperintensity volume and smaller whole-brain and hippocampal volumes in later life (69–71 years), a finding that was unrelated to cognitive performance assessed with the Preclinical Alzheimer Cognitive Composite, nor with cerebral amyloid-β load [13]. Specifically, 10 mmHg greater systolic and diastolic BP at 53 years of age were associated with a relative increase in mean white matter hyperintensity volume of 7% and 15%, respectively [13]. 

In terms of late exposure to hypertension, among 1440 Framingham Offspring participants who were free of dementia at midlife (mean age: 55 years) and were followed-up over an 18-year exposure period, midlife systolic hypertension (≥140 mmHg) and its persistence into late life were associated with an increased dementia risk in late life by 57% to 96%, respectively [14]. In the same study, people in late midlife (mean age: 62 years) with a healthy cardiovascular profile (current non-smokers on a healthy diet reporting regular physical exercise, with BP < 120/80 mmHg, normal body mass index (BMI), and normal glycolipid profile) had a 20% less risk of developing Alzheimer-type dementia and half the risk of developing vascular dementia in the following 10 years [14]. A retrospective analysis of the Health and Retirement Study (HRS), enrolling a US nationally representative panel of individuals aged 50 and over, showed that hypertension was associated with reduced life expectancy free of cognitive impairment (−4.08 years at the age of 55; −3.38 years at the age of 65; −2.39 years at the age of 75; and −1.34 years at the age of 85) [15]. Having multiple cardiovascular risk factors and diseases (hypertension, diabetes, heart disease, and stroke) was associated with an exponential decrease in the proportion of the lifespan lived in good cognitive health [15].

Mechanistically, high BP can affect cognitive performance through several pathways. Hypertension is a powerful risk factor for atherosclerosis and the most relevant modifiable risk factor for stroke, a condition that exacerbates the risk for both vascular and non-vascular dementia [8]. In addition, persistent exposure to pulsatile stress promotes microvascular damage with lipohyalinosis that results in white matter damage, lacunas, brain atrophy, and loss of cortical connections, thus affecting cognitive performance [8]. Consistent with this, spontaneously hypertensive rats (SHR) show evidence of cerebrovascular changes, brain atrophy, loss of nerve cells in cerebrocortical areas, and glial reaction, similar to what can be found by in vivo imaging studies in essential hypertensive individuals [16]. In parallel, local or diffuse hypertension-induced alterations in cerebral perfusion with cerebral hypoxia, oxidative stress, and endothelial dysfunction, are thought to contribute to the impairment of neuronal metabolism and neurotransmission, which act as possible triggers for the neuronal inflammatory and degenerative phenomena underlying the development of Alzheimer’s disease [8]. 

A recent proof of the neuroprotective effects of hypertension control comes from the results of the Systolic Blood Pressure Intervention Trial (SPRINT) Memory and Cognition in Decreased Hypertension (MIND) sub-study, which assessed the occurrence of all-cause adjudicated probable dementia, mild cognitive impairment, or their composite in relation to standard (<140 mmHg) or intensive (<120 mmHg) systolic BP lowering for a mean total follow-up of almost 6 years. Intensive systolic BP lowering was associated with a significant reduction in the risk of mild cognitive impairment (14.6 vs. 18.3 cases per 1000 person-years; HR: 0.81, 95% CI: 0.69–0.95) and with a non-statistically significant reduction in all-cause probable dementia (7.2 vs. 8.6 cases per 1000 person-years; HR: 0.83; 95% CI 0.67–1.04), for a global risk reduction of 15% [17]. In agreement with this, in the relatively young cohort of 7063 participants in the Estudo Longitudinal de Saúde do Adulto (ELSA Brasil) (mean age at baseline: 58.9 years), who were followed-up for 4 years, noncontrolled hypertensive individuals had significantly faster declines in memory and global cognitive function performance than controlled individuals [18], with no effect of hypertension treatment per se on cognitive function. Besides daytime BP profile, nighttime BP is also emerging as a relevant contributor to cardiovascular health and disease. Consistent with this, poor nocturnal BP control, expressed as reverse dipping, appears to have a relevant role in hypertension-associated small vessel cerebrovascular disease and related memory impairment [19]. A particularly detrimental effect of reverse dipping on cognitive function has been described in elderly adults [19,20].

Based on the available evidence, a recent report on dementia prevention, intervention, and care states that antihypertensive treatment is the only known effective preventive medication for dementia and that maintaining systolic BP ≤ 130 mm Hg in midlife from the age of 40 years is recommended to reduce the risk of incident dementia [6]. 

## 3. Hypotension and Cognitive Function

**Figure 1:**
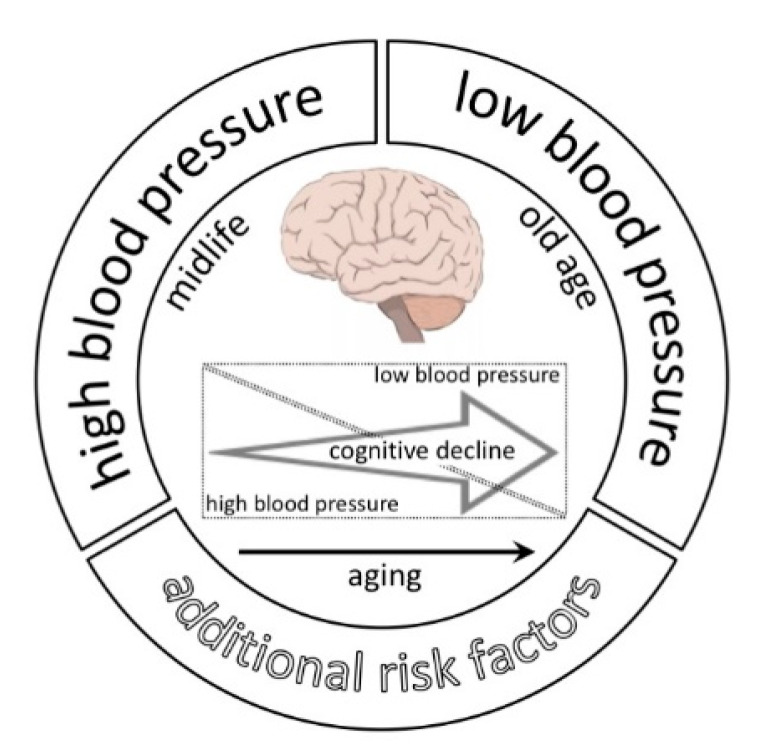
Relationship between BP and cognitive decline. The relative impact of high BP on cognitive dysfunction progressively declines with aging, while the influence of low BP progressively increases. The prolonged exposition to additional cardiovascular risk factors further contributes to the onset and progression of cognitive dysfunction.

In parallel with the deleterious impact of high BP, cerebral hypoperfusion secondary to hypotension and to excessive BP variability can also exert detrimental effects on neurocognitive fitness [20,21] (Figure 1). With progressive ageing, BP regulation capacity gradually declines. Observational evidence in centenarians indicates that higher systolic BP and higher mean arterial pressure are associated with better mental and physical performance as well as with increased survival [22]. In contrast, low systolic BP in the older individual (>85 years) predicts the onset of dementia [23]. Sex-based studies are lacking on this topic, but the available evidence suggests the so-called male-female health-survival paradox, according to which women have a survival advantage, but in which centenarian men usually benefit from better physical and cognitive health than centenarian women [24]. While this finding may be confounded by bias in survey, with men being more reluctant than women to participate or accurately report if they have disabilities or diseases, it is likely that multiple causes are involved, including fundamental biological and behavioral differences between the sexes, such as genetic features, immune system reactivity, hormones, as well as treatment seeking and compliance [24].

Data from the Leiden 85-plus Study showed an association between accelerated cognitive decline and lower BP in the oldest old (>85 years) taking antihypertensive medications (*n*. 249), with an annual mean change in the Mini-Mental State Examination (MMSE) score of −0.35 points (95% CI: −0.60 to −0.11, *p* = 0.004) per 10 mmHg lower systolic BP, a finding that was not replicated among untreated individuals (*n*. 321) [25]. Similarly, in a cohort study on 172 hypertensive outpatients with overt dementia and mild cognitive impairment (68% and 32%, respectively; mean baseline MMSE score: 22.1 ± 4.4; mean age: 79 ± 5 years; median follow-up: 9 months), low daytime systolic BP assessed by ambulatory BP measurement (ABPM) (≤128 mmHg) was associated with significantly greater worsening of the MMSE score (mean: −2.8 ± 3.8) compared with intermediate (129–144 mmHg) and elevated (≥145 mmHg) daytime systolic BP values among individuals receiving antihypertensive medications, but not in the untreated counterparts [21]. Despite the short follow-up time, these findings add to the sparse literature on the underrepresented category of cognitively impaired hypertensive patients, who are usually excluded from randomized clinical trials, and suggest a possible preferential approach to the management of hypertension in this category based on ABPM. 

Recently, results from the SPRINT MIND (*n*. 9361, of which 28.2% were aged ≥75 years) indicated that, compared with individuals with orthostatic hypotension treated to a systolic BP target of <140 mmHg (*n*. 340), the risk of dementia did not increase among those with orthostatic hypotension treated to a systolic BP target of <120 mmHg (*n*. 345) after a median intervention period of 3.34 years and an overall median follow-up of 5.11 years [17]. Conversely, data from 3121 patients aged ≥80 years enrolled in the Hypertension in the Very Elderly Trial (HYVET) showed that orthostatic hypotension, defined as a decrease of at least 15 mmHg in systolic or 7 mmHg in diastolic BP from sitting to standing, was associated with a 36% increased risk of cognitive decline (HR: 1.36, 95%: CI 1.15–1.59), as well as with a 34% higher risk of incident dementia (HR: 1.34, 95% CI: 0.98–1.84) [26]. The risk was higher in the presence of subclinical orthostatic hypotension (i.e., a symptomatic fall in systolic BP of <15 mmHg upon standing or in diastolic BP of <7 mmHg upon standing), with a 56% increased risk of cognitive decline and a 79% increased risk of dementia [26]. The exact mechanisms behind these observations are unknown, but they are likely to result from a progressive failure in the autonomic function and other homeostatic mechanisms attributable to vascular stiffening and atherosclerosis in the oldest old population. 

In agreement with this is the observation of an increased risk of developing all-cause dementia, vascular dementia, and Alzheimer-type dementia in association with increased day-to-day BP variability (coefficient of variation, CoV) among 1674 community-dwelling Japanese elderly individuals (≥60 years of age) of the Hisayama study [27]. Increased BP variability can mirror excessive orthostatic BP drops, possibly amplified by arterial stiffness, or repeated periods of hypotension. The consequent hemodynamic instability may increase shear stress, leading to small vessel disease, cerebral hypoperfusion, and neurodegeneration. The risk of Alzheimer-type dementia was significantly higher in subjects with elevated BP variability (CoV: ≥7.60% for systolic BP, ≥7.61% for diastolic BP) compared with individuals with less BP variability (CoV: ≤5.07% for systolic BP, ≤4.83% for diastolic BP), regardless of absolute BP values. This finding may reflect central cholinergic dysfunction observed in individuals with prodromal Alzheimer disease and consequent impairment in the mechanisms of autonomic regulation of BP.

In agreement with this, a decrease in BP levels appears to occur after the onset of dementia, probably as a consequence of brain lesions on the mechanisms of BP regulation [28,29]. The additive role of extra-cardiovascular drugs that can cause hypotension, such as certain anti-parkinsonian, antidepressants, and antipsychotic drugs of common use in the geriatric patient, should not be discarded [3].

## 4. Antihypertensive Drugs: Benefits and Pitfalls

A recent report on dementia prevention, intervention, and care states that treatment for hypertension is the only known effective medication strategy to prevent dementia [6]. This statement is grounded on the convergent results of four meta-analyses suggesting a reduced risk of dementia in individuals treated with antihypertensive medications compared with no use [30,31,32,33].

Pooling the data from eight completed randomized controlled trials that assessed the impact of different approaches to BP lowering on incident dementia showed a nonsignificant 7% lower risk of the outcome among individuals in the intervention arm (relative risk, RR: 0.93, 95% CI: 0.86–1.00, *p* = 0.07; I^2^ = 0%). When limiting the analysis to those trials that achieved a large (≥10 mmHg) systolic BP difference, a potential dose response was observed, with 12% less risk of incident dementia associated with lower achieved BP (RR: 0.88, 95% CI: 0.78–0.98; *p* = 0.03; I^2^ = 0%) [17,32,34,35,36].

A meta-analysis of six prospective community-based studies (*n* = 31,090 dementia-free adults aged 55 years and older) with median cohort follow-up of 7 to 22 years showed a 12% reduced risk for dementia (HR: 0.88, 95% CI: 0.79–0.98) and 14% lower risk of Alzheimer disease (HR: 0.84; 95% CI: 0.73–0.97) in association with antihypertensive medications use compared with nonuse among individuals with high baseline BP (≥140/90 mmHg; *n* = 15,537) [33]. The authors did not find any evidence of a drug-specific effect on the reduction of dementia risk [33]. In contrast with this, an earlier meta-analysis of 15 prospective, longitudinal, and database registry studies (*n* = 52,599 adults without dementia, median age at enrollment 76.1 years) showed that diuretic use compared with nonuse or with other antihypertensive drugs was associated with a 17% reduced risk of dementia (HR: 0.83; 95% CI: 0.76–0.91, *p* < 0.0001, I^2^ = 0%) and an 18% lower risk of Alzheimer’s disease (HR: 0.82; 95% CI: 0.71–0.94, *p* = 0.004, I^2^ = 0%) after a median follow-up of 6.1 years [30]. However, the likelihood of prescription or indication bias was acknowledged, as was the possibility that the observed findings could be attributable to effective BP lowering with diuretics rather than to drug-specific mechanisms [30]. Similarly, a meta-analysis of 10 prospective studies (*n* = 75,239 elderly hypertensive individuals ≥ 60 years without baseline dementia; median age 72.2 years; median follow-up time 8.21 years) showed that the use of calcium channel blockers was associated with 30% reduction in the risk of developing dementia (RR: 0.70, 95% CI: 0.58–0.85, *p* = 0.0003) compared with nonuse [31]. The hypothetical biological explanation for these findings relates to the inhibition of calcium ions entry in the neuronal cells, hence protecting against redox unbalance, cellular death, and possibly against increased amyloid beta production that triggers neurodegeneration in Alzheimer’s disease [2,37,38]. Consistent with this, dihydropyridine-type calcium channel blockers were described as the drug class with the most documented protective effect on hypertensive brain damage in animal models of hypertension [16]. However, animal studies assessing the relationship between antihypertensive medications and cognitive function often lack information on BP values [39]. 

There is also proof of concept that ACE-inhibitors and angiotensin receptor blockers exert a protective effect on cognition, in that they can downmodulate the pro-inflammatory effect of the activated renin-angiotensin system, reduce oxidative stress, and counteract the local effects of Ang II, namely vasoconstriction and acetylcholine release inhibition [2]. Nevertheless, a recent meta-analysis of 27 prospective longitudinal studies and BP trials with data on antihypertensive class (*n* = 50,000 participants) did not find evidence of neuroprotective effects in association with specific antihypertensive drug classes (calcium channel blockers, ACE-inhibitors, angiotensin receptor blockers, beta-blockers, or diuretics) among individuals aged ≥65 years and followed for at least 5 years [40]. Data on younger participants were sparse and inconclusive [40]. Another worth-mentioning consideration relates to the interaction reported for ACE-inhibitors with the apolipoprotein E4 (ApoE4) allele. ApoE4 is considered a major genetic risk factor for late-onset and sporadic Alzheimer disease and incident memory decline, and there is evidence that ACE inhibitors are protective against the risk of Alzheimer’s disease in the absence of ApoE4, but not in the presence of the ApoE4 allele. The hypothetical mechanisms behind the unfavorable combination include a synergistic effect in reducing the clearance of amyloid-β peptide (Aβ), or the detrimental cerebrovascular effects of excessive angiotensin II [41,42].

The studies that assessed the impact of BP lowering on cognitive performance suffered from intrinsic limitations affecting subsequent pooled analyses, e.g., the predominantly old age of participants at enrollment, their heterogeneous sociodemographic background, variability in their clinical features that was strictly related to the widespread primary focus on cardiovascular outcomes, and the infrequent evaluation of cerebrovascular pathology with advanced technologies [32]. Consequently, limited advancement was gained in the understanding of the pathogenic relation of BP with incident dementias, and more focused evidence is required from future studies.

## 5. Future Perspectives

The current knowledge gaps in the relation of BP profile with cognitive performance can only be filled by specifically oriented, multimodal research. At the molecular level, it remains undetermined whether high BP elicits specific neurohormonal responses in different brain areas and how it interacts with key pathogenic factors involved in neurodegeneration such as tau and Aβ. New and available experimental models can be exploited to investigate these aspects. In parallel, combining brain imaging approaches with advanced statistical methodology, including artificial intelligence and machine learning, can maximize the information on the anatomic and functional aspects of neurodegeneration in hypertension. For a powerful use of such techniques, data need to be generated from sufficiently powered, long-lasting, inclusive trials, in which all the relevant variables in the relation (e.g., race/ethnic groups, comorbidities, and medications) are well-represented, thus allowing informative subgroup analyses, and in which the methodology for the assessment of BP and cognitive performance are reliable and reproducible. Similar trials need to span a wide range of ages and include sex-based analyses in order to define the temporal features of the BP-cognition relation in women and men.

## 6. Conclusions

Epidemiological evidence consistently indicates that high BP, particularly in midlife, is a risk factor for the development of dementia in late life. Combined data from BP lowering trials suggest a benefit of effective BP control on cognitive performance, with possible ideal timing for initial BP monitoring and interventions at around 40 years of age to maximize late-life brain health [6]. In parallel, antihypertensive treatment needs to be finely calibrated to avoid hypotension (e.g., excessive orthostatic BP fall, excessive BP variability), given its unfavorable effect on cognitive performance, as well as on highly prevalent clinical manifestations of the geriatric patients that can have an impact on individual global fitness, namely depression and delirium. Despite the lack of a clear drug class superiority from the available clinical evidence, putative specific benefits have been described for calcium channel blockers, ACE-inhibitors, and sartans in basic research studies, altogether supporting their preferential use in the old hypertensive patient against cerebrovascular events and cognitive impairment [43].

All the unanswered questions in the relation of BP with dementia need testing in specifically designed and technologically advanced studies where the underlying pathophysiological mechanisms are definitively clarified, allowing for a tailored preventive, diagnostic, and therapeutic approach to one of the most widespread, yet indecipherable, medical challenges of the present day and the upcoming future.
